# Disordered Binding Regions and Linear Motifs—Bridging the Gap between Two Models of Molecular Recognition

**DOI:** 10.1371/journal.pone.0046829

**Published:** 2012-10-03

**Authors:** Bálint Mészáros, Zsuzsanna Dosztányi, István Simon

**Affiliations:** Institute of Enzymology, Research Centre for Natural Sciences, Hungarian Academy of Sciences, Budapest, Hungary; University of Toronto, Canada

## Abstract

Intrinsically disordered proteins (IDPs) exist without the presence of a stable tertiary structure in isolation. These proteins are often involved in molecular recognition processes via their disordered binding regions that can recognize partner molecules by undergoing a coupled folding and binding process. The specific properties of disordered binding regions give way to specific, yet transient interactions that enable IDPs to play central roles in signaling pathways and act as hubs of protein interaction networks. An alternative model of protein-protein interactions with largely overlapping functional properties is offered by the concept of linear interaction motifs. This approach focuses on distilling a short consensus sequence pattern from proteins with a common interaction partner. These motifs often reside in disordered regions and are considered to mediate the interaction roughly independent from the rest of the protein. Although a connection between linear motifs and disordered binding regions has been established through common examples, the complementary nature of the two concepts has yet to be fully explored. In many cases the sequence based definition of linear motifs and the structural context based definition of disordered binding regions describe two aspects of the same phenomenon. To gain insight into the connection between the two models, prediction methods were utilized. We combined the regular expression based prediction of linear motifs with the disordered binding region prediction method ANCHOR, each specialized for either model to get the best of both worlds. The thorough analysis of the overlap of the two methods offers a bioinformatics tool for more efficient binding site prediction that can serve a wide range of practical implications. At the same time it can also shed light on the theoretical connection between the two co-existing interaction models.

## Introduction

Most proteins carry out their function through recognizing and binding to partner molecules, a vast majority of which are proteins themselves. Studying the details of this molecular recognition process is essential to understand the organization of living cells [1]. Following the appearance of the first protein structures the dominant view of protein-protein interactions was represented by interactions between well-folded domains, where two protein domains interact as a result of steric, hydrophobic and charge complementarity of their interacting surfaces [2]. However, later it was recognized that a large fraction of protein-protein interactions are not mediated exclusively by folded domains. Interactions involving non-folded domains represent a distinct type of binding mode that is prevalent in various regulatory and signaling processes [3]–[6]. In this work we study the relationship between two related concepts that were introduced to describe such interactions: disordered binding regions and linear interaction motifs.

Disordered binding regions correspond to functionally relevant interaction sites residing in intrinsically disordered proteins and protein regions (IDPs and IDRs). The characteristic feature of these regions is that in isolation they exist as ensembles of rapidly interconverting conformations. Upon recognizing their partner molecule, they can undergo a disorder-to-order transition and adopt a well-defined conformation. This coupled folding and binding process results in a lowered binding strength making such regions ideal for low affinity, weak and transient binding, crucial for regulatory and signaling pathways [5], [7]. Furthermore, the plasticity of these regions provides them with increased interaction capacity. The specific properties of such binding regions are largely responsible for the biological significance of disordered proteins [8], [9]. Disordered proteins are often found among hubs of protein-protein interaction networks [4], [10], [11] and can play a major role in the evolutionary adaptability of interactomes providing possible points of network re-wiring [12]. The analyses of genomic sequences revealed that protein disorder is prevalent and increases with evolutionary complexity [13], [14]. Specifically, about 50% of human proteins are predicted to contain at least one larger disordered region, and it was shown that the primary reason for the emergence of these regions is to harbor binding sites [15]. Recognizing the importance of protein disorder, especially in critical processes such as transcription, translation, regulation, signal transduction and stress-response of higher eukaryotes [5], [16]–[19], fuelled the study of such interactions.

Available structures of disordered binding regions in their bound form showed very distinct properties compared to complexes of globular proteins [20], [21]. Disordered binding regions adopt a largely extended conformation on the surface of their partner molecule. Furthermore, these disordered binding sites are usually well localized in the sequence in a sense that the residues involved in the interaction can be mapped to a single continuous segment of the protein. This is in contrast to binding sites of globular proteins that are formed by distant regions of the polypeptide chain brought together only as a result of folding [20]. Disordered binding regions are also usually more hydrophobic than their sequential neighborhood [20], [21]. The specific sequence properties of these regions enable their recognition from the amino acid sequence. The first dedicated prediction methods, α-MoRF-Pred [22] and α-MoRF-PredII [23] targeted regions adopting an alpha helical conformation upon binding. In contrast, ANCHOR [15] is generally applicable regardless of the bound structure of the binding site and currently is the only such method that is publicly available [24]. This method is based on the modeling of the physical background of such binding processes using statistical potentials. Through a simplified model, ANCHOR can capture the disordered nature of these binding regions that dominates their isolated state, as well as the driving force of the binding. The strength of ANCHOR apart from its generality lies in that the structural context is inherently modeled during the prediction. As the relatively high success rate of ANCHOR (around 70%) indicates, this is essential for recognizing most of the biologically occurring disordered binding regions. Nevertheless, ANCHOR is only able to predict aspecific interaction sites without providing information about the interacting partner.

Parallel to the disordered binding region concept, interactions between short regions of proteins and globular domains have been extensively studied using the concept of linear motifs [25]. It was observed, that the binding to certain globular domains – such as SH2/SH3, 14-3-3, WW and kinase domains – is mediated by a limited number of residues that can be represented by either a sequence logo or a regular expression [26], [27]. The linear motifs capture the sequence features shared among usually non-homologous interacting partners [3], [26]. These features encompass fixed residues common to all interacting partners, interspersed with flexible positions that can accommodate a variety of amino acids without disrupting the binding. These motifs can be found dominantly in eukaryotic proteins, however some of these motifs can be expected to be present in other domains of life, and even in viruses. The most comprehensive and extensive available database of these motifs is the Eukaryotic Linear Motif (ELM) database [27]. Motifs are categorized into four groups: cleavage sites (CLV), ligand binding sites (LIG), targeting signals (TRG) and modification sites (MOD). The current update of the ELM database comprises 1800 annotated motif instances representing 170 distinct motif classes [27]. The present collections is expected to contain only a small proportion of possible motif mediated interactions, as a recent moderate estimate places the number of individual, motif mediated interactions in the human proteome above 35,000 [28].

The basic assumption behind the concept of linear motifs is that these sites function autonomously, largely independent of the other regions of the protein they are embedded in. For interactions mediated through such motifs, basic pattern matches can be used to identify putative binding partners of a given domain in unknown sequences. The strength of this method besides its simplicity is that it automatically gives information about the possible interacting partner. However, these patterns can arise purely by chance with a relatively high probability [29], resulting in a massive amount of false positive hits by naïve motif searches. This is partially the consequence of the incomplete description sequence patterns have to offer. Inside a living cell, the functionality of the motifs is modulated by spatial and temporal control [6]. However, the insurance of the biological relevance of the binding also requires the description of the proper structural context of the motif, such as being accessible, flexible and capable of forming the secondary structure necessary to fit into the binding cleft of the target domain. Unfortunately, current motif definitions do not include such information.

The disordered binding region and the linear motif concepts describe molecular interactions on different bases: the former focusing on the structure (or the lack and formation of it) and the latter approaching the problem through the sequence. However, the interactions described by the two concepts share a high degree of similarity. In both cases the interaction is confined to a relatively short, linear sequence region in one of the partners. Additionally, many known linear motif instances were shown to reside in disordered protein regions. Often the same interaction was categorized as an example of both linear motif mediated binding and of disordered binding regions, such as the binding of p53 to MDM2 and the N-terminal region of p27 binding to the cyclin A-CDK2 complex. Through many common examples, both the binding of disordered proteins and linear motifs have been shown to be essential for the integration and propagation of regulatory signals controlling eukaryotic cell physiology [6]. Furthermore, both models fit well with the description of molecular switches controlled by various post-translational modifications [30], [31], localization or competitive binding [32]. The interplay between protein disorder and motif regulation has been also shown at a systems level with regard to the organization and regulation of living cells [33]. This, together with the realization that in many cases disordered binding regions and linear motifs describe the same interactions has led to studies where the two concepts and hence the two terms are essentially used interchangeably, both in biological considerations [32] and technical applications [34].

In spite of the fact that these two interaction models have intertwined at the anecdotal level, the systematic study of their connection has not been directly assessed. Since the number of experimentally verified examples are rather limited in both bases, their connection at a large scale can be studied only via bioinformatics approaches. The present work investigates this connection through two prediction methods, each tailored specially for identifying the respective type of interaction sites. Disordered binding regions are identified by ANCHOR and linear motif searches are carried out by using regular expressions taken from the ELM database. Through the overlap of these two approaches we set out to take the next step in the integration of the two concepts.

## Results

### Predictive Power of Linear Motifs

One of the main limitations of using linear motifs in the prediction of protein-protein binding regions is the weak definition of the motifs. The vast amount of false motif hits emerging in simple motif searches can be qualitatively demonstrated through biological considerations.

**Figure 1:**
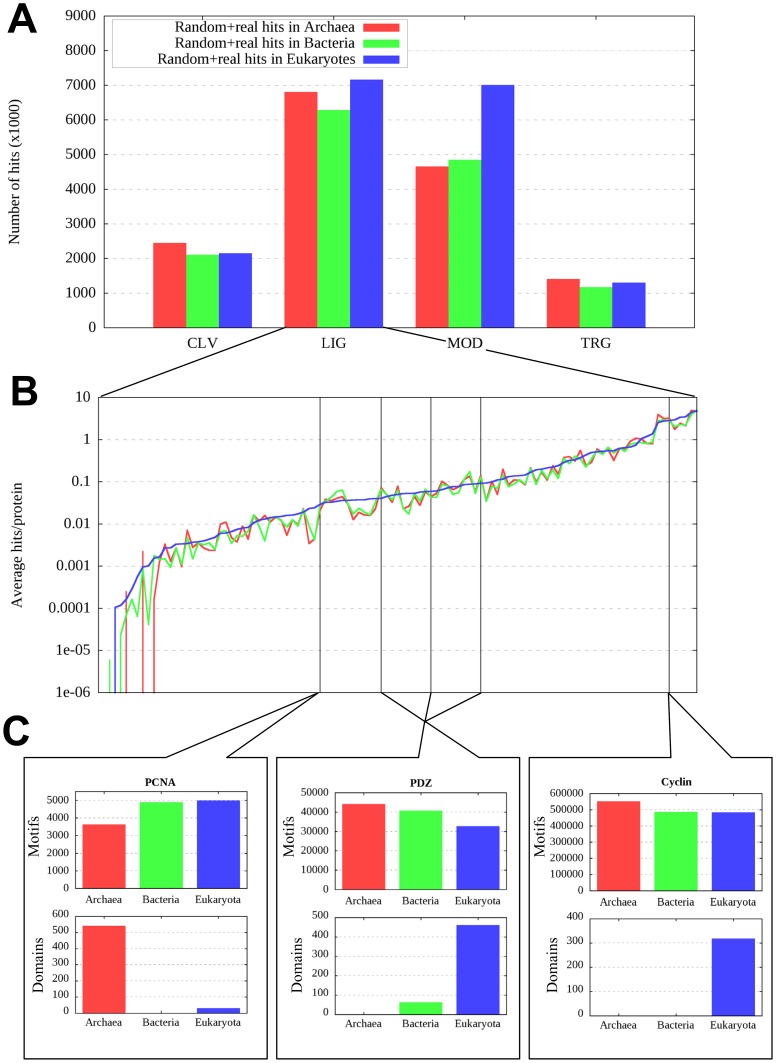
Results of motif scans in the three domains of life. A: the number of found motif hits from the four different motif groups (CLV – cleavage sites, LIG – generic ligand binding motifs, MOD – modification sites, TRG – target signals) in the eukaryotic (blue), bacterial (green) and archaeal (red) proteins included in the UniProt database. As the size of the three databases are different, the number of actual hits in the prokaryotic sets were scaled with the ratio of the number of residues in each dataset. B: The average number of motif hits per protein for the three databases covering the three domains of life. Again, hit numbers in prokaryotic sets are corrected for different number of residues compared to the eukaryotic dataset. Coloring is identical to that of part A (red – archaea, green – bacteria, blue – eukaryotes). C: The upper bars show the number of found hits in the three domains of life for PCNA, PDZ and cyclin binding motifs (the average hits per protein for the three motifs are shown with vertical lines in part B; note that there are three different PDZ binding motifs and each one is shown with separate lines in part B but only their cumulative numbers are shown in part C). Lower bars show the actual number of corresponding partner domains that can serve as interaction partners for these motifs in the same datasets. Domain occurrences were taken from the PFAM database. Prokaryotic hit numbers are corrected for different number of proteins and the coloring scheme follows that of parts A and B.

For this purpose, the motifs collected in the ELM database were used. As these motifs were described mostly in eukaryotes, there should be a strong bias of real occurrences to appear in eukaryotic proteins as opposed to bacterial and archaeal proteins. In contrast to this, scanning bacterial and archaeal protein datasets (see Methods) for ELM motif pattern matches yields hit numbers comparable to that of searches in eukaryotic proteins (see figure 1A). These hit numbers include both real instances and false positive (random) hits. Although the ratio of true and random hits is unknown, real hits are expected to show a pronounced enrichment in eukaryotes. On the other hand, random occurrences are expected to appear with approximately the same frequency in all three domains of life. The lack of difference between eukaryotes and prokaryotes in this regard is the most alarming in the case of target (TRG) motifs (controlling sub-cellular localization of proteins), as the lack of cell compartments in prokaryotes makes such a widespread biologically relevant usage of target signals very improbable.

Focusing on generic ligand binding motifs (LIG), figure 1B shows that the number of matches for these motif patterns from the three domains of life are mostly indistinguishable even when assessed for each motif separately. Some well defined motifs – such as the GYF domain binding motif – have pattern descriptions that match only a handful of protein sequences (18 out of all eukaryotic sequences from SwissProt and none of the archaeal or bacterial sequences). However, this is rather the exception than the rule, with nearly 76% of the LIG motif patterns matching at least 1 out of 100 proteins. These motifs cover a wide range of functions such as the interaction with 14-3-3, WW, PDZ, PCNA domains, nuclear receptors and even the interaction with MDM2 via a motif that is experimentally described exclusively in the p53 protein family. Considering the biological meaning of these motifs, it is clear that with a few exceptions, naïve motif searches are dominated by false positives.

Ligand binding motifs mediate interaction with a well defined protein partner domain. The occurrence of three example LIG motifs are shown in figure 1C. The top part of figure 1C shows the occurrence of PCNA, PDZ and cyclin binding motif hits (random+real occurrences). The position of these three motifs are shown in figure 1B with vertical lines (note that there are three sub-types of PDZ motifs and in figure 1C the occurrence of all three types are added). The bottom parts of figure 1C show the occurrence of the corresponding interacting domains in the three domains of life. The occurrence of PCNA, PDZ and cyclin domains is highly unbalanced with PCNA domains being absent in bacteria, PDZ domains being absent in archaea and cyclin domains being exclusive to eukaryotes. The presence of real motifs is linked to the presence of the interacting partner domain, however, the corresponding motif hits do not reflect these specific distributions and all three motif patterns can be found ubiquitously in all three domains of life.

The same overprediction trend can be shown for targeting signals as well. Scanning the human proteome (see Methods) for TRG motifs, about 92% percent of the proteins match motifs that – in biologically active form – are exclusively found in membrane proteins (TRG_ENDOCYTIC_2, TRG_ER_diArg_1, TRG_ER_diLys_1 and TRG_LysEnd motifs). Furthermore, 41% of human proteins match classical nuclear localization signals and 33% are predicted to be localized to the peroxisome. The irrationally high numbers for these localizations and the large overlap between incompatible localizations (95% of proteins matching NLS’s also match membrane localization motifs) show that targeting motifs suffer from the same under-definition as ligand binding motifs.

### Combining Linear Motif and Disordered Binding Region Predictions

#### Overall efficiency and the reduction of false positives

**Figure 2:**
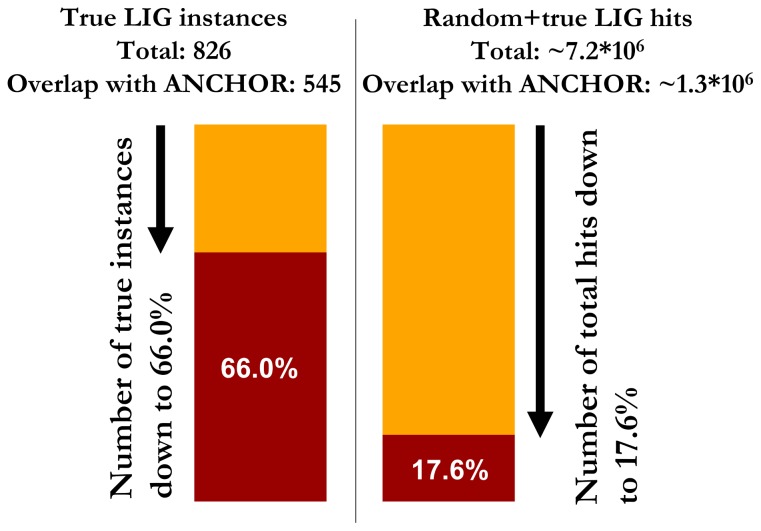
The predictive power of ANCHOR as a filter in motif searches. Left: fraction of known instances of ligand binding motifs recognized by ANCHOR. Right: the reduction in the number of ligand binding motif hits in the eukaryotic sequences of UniProt.

The overlap between predicted disordered binding regions and linear motifs was tested using ANCHOR predictions and annotated ligand binding linear motif (LIG) instances from the ELM database. For this purpose a more permissive version of ANCHOR was chosen, where the prediction threshold was reduced to 0.4 instead of the original 0.5. Motif instances were checked and filtered for similarity to minimize redundancy (see Methods). The majority of annotated LIG motif instances were recognized by ANCHOR as binding regions yielding a recovery rate of 66%. In contrast, the overlap between ANCHOR predictions and unfiltered motif pattern matches in the eukaryotic sequences in UniProt (containing both random and true motif instances) is significantly lower with 17.6% (see figure 2). In total 7,164,890 LIG motif hits were found in the total of 171,208 sequences. Upon filtering the hits with ANCHOR, only 1,262,532 LIG motif hits remained, yielding a reduction of over 82%. The large difference between the overlap of ANCHOR with true and true+random motif occurrences shows ANCHOR’s sensitivity to true linear motif instances.

**Figure 3:**
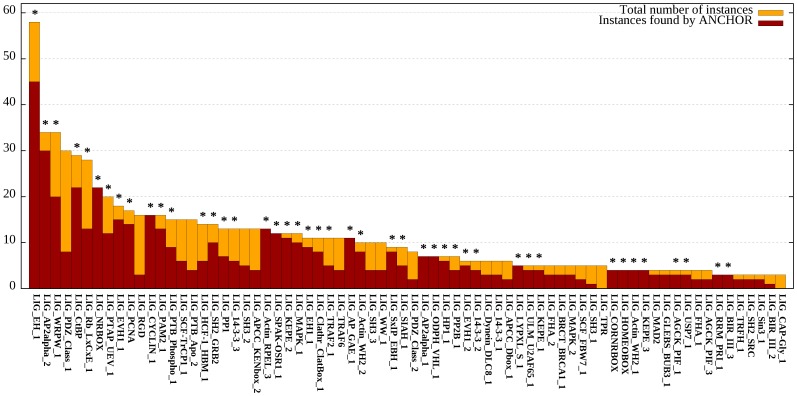
Efficiency of ANCHOR for individual LIG motifs. The total number of annotated instances for each of the ligand binding motifs that have at least three independent instances in the ELM database. Dark red bars show the number of instances overlapping ANCHOR predicted binding regions. Stars mark the motifs for which the recovery rate is significantly higher than that expected by chance alone (see Methods).

ANCHOR’s recovery rate and the reduction of hits, however, is highly uneven between different motifs. At one extreme, all 22 instances of the nuclear receptor box motif (LIG_NRBOX) were recognized, and at the other, none of the 5 TPR binding motifs were found. To give a more detailed picture on the efficiency of ANCHOR in motif recognition, recovery rates and the reduction of hits (calculated on the eukaryotic sequences in UniProt) were calculated for each motif separately. Figure 3 shows the total number of true instances and the number of these overlapping with ANCHOR predictions for all LIG motifs that had at least three independent annotated instances. For each motif the rate of recovery was compared to the random overlap (see Methods). For motifs marked with asterisk the overlap is significantly higher than expected from random.

**Figure 4:**
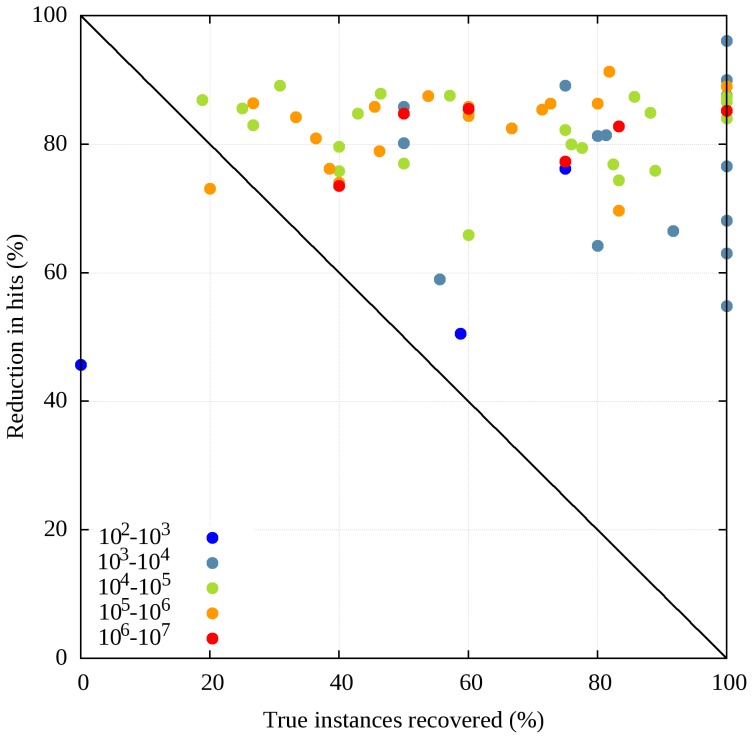
Recovery ratio and reduction in total number of hits. Ratio of true instances recovered by ANCHOR versus the reduction in the number of total hits in the eukaryotic sequences of UniProt as a result of ANCHOR filtering for all ligand binding motifs that have at least one annotated instance in the ELM database. Colors show the order of magnitude of the number of hits. The diagonal line marks the expected performance of a random filtering procedure.

The true instance recovery and the reduction of hits is shown in figure 4 for LIG motifs. Motifs are color-coded according to the order of magnitude of the number of hits they produce when scanning the eukaryotic sequences of UniProt. It can be seen that for well defined motifs giving a moderate number of hits (<10^4^) the reduction rate is lower with an average of approximately 60%. However, for more ill-defined motifs (>10^4^ hits), the reduction rate increases and reaches approximately 85%. This shows that ANCHOR can be especially useful for filtering hits of poorly defined motifs, whereas for well-defined motifs the definition already guarantees a more moderate false positive rate. The overall performance of ANCHOR in motif recognition is also reflected in figure 4: with a random filtering procedure the points describing performance on individual motifs should lie on the marked diagonal. With a few exceptions, ANCHOR filtering is on the upper right side of the diagonal.

#### Efficiency by structural context

The efficiency of ANCHOR is highly dependent on the structural context of the motif instance. Although most motif instances can be found in disordered protein regions, some motifs are known to reside in globular domains in accessible surface loops. Furthermore, some motifs are generally found at terminal regions of proteins. For example, the PDZ motifs occur exclusively at the C-terminus of proteins and are usually preceded by a folded domain. As ANCHOR relies heavily on the disordered state of the protein region to recognize disordered binding regions, in these cases its efficiency is expected to be lower.

To test this, true LIG motif instances were grouped according to the disorder or order of the sequence regions flanking the instance. Based on this, three groups were established. A motif instance is categorized as disordered, if both the N- and C-terminal flanking regions are predicted to be disordered by IUPred [35], [36]. Mixed instances are flanked by a disordered region on one side and by an ordered one on the other side. Ordered instances reside in a sequential environment fully predicted to be ordered.

**Figure 5:**
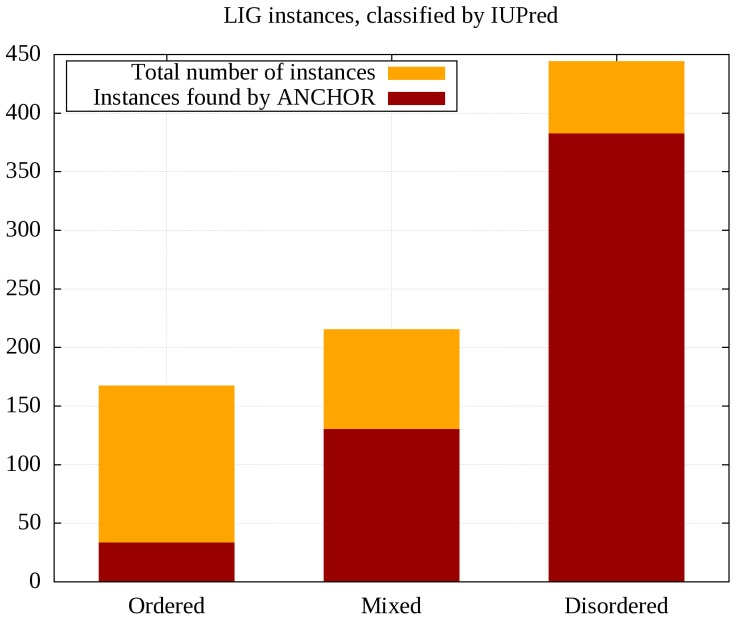
Efficiency of ANCHOR on linear motifs with respect to structural context. Instances are classified according to the predicted disorder status of their flanking sequential environment. Motif instances with both N- and C-terminal flanking regions predicted by IUPred as ordered are classified as ‘Ordered’, instances with one or both flanking regions predicted to be disordered are classified as ‘Mixed’ or ‘Disordered’, respectively.

Figure 5 shows the efficiency of ANCHOR on all three groups. This efficiency varies highly between the groups. Only 19.7% of ordered instances are found, but the recovery rate increases to 60.5% and 86.0% for mixed and disordered instances, respectively. These results are largely independent of the prediction method used for the assignation of disorder status, and remained consistent upon using DISOPRED2 or VSL2 (data not shown).

#### Efficiency by adopted secondary structure

Although the majority of true motif instances reside in disordered protein regions, upon binding to the target domain these sequence regions generally adopt a stable structure. This is exemplified by the solved structures of many motif instances bound to their partners. As the molecular details of the binding process are reflected in the resulting structure, the efficiency of ANCHOR can vary among motifs with different adopted secondary structures.

To investigate this, bound structures of known true instances were analyzed. The bound complex structures were collected from the ELM database and additional structures were identified using similarity searches between the sequences of the proteins containing the instances and the PDB (see Methods). It was shown that the bound structures of various instances of the same motif are highly similar [37], therefore it is plausible to assume that the bound secondary structure of a motif instance is representative of the whole motif class. As the bound regions are generally short, they can be characterized by a single dominant secondary structure. In this study we used the three standard secondary structure assignments of helix, extended (β strand) and coil. However, as a significant class of motifs (such as EVH1, SH3 and certain WW motifs) are proline rich, a fourth structural class of poly-proline II helices was added. A list of instances with known bound structures are shown in table S1. Based on the notion that various instances of the same motif adopt very similar bound structures, we extended the secondary structure definitions and motifs that have at least one annotated instance with an available bound structure themselves were assigned a secondary structure category. These motif level structure definitions are shown in table S2.

**Figure 6:**
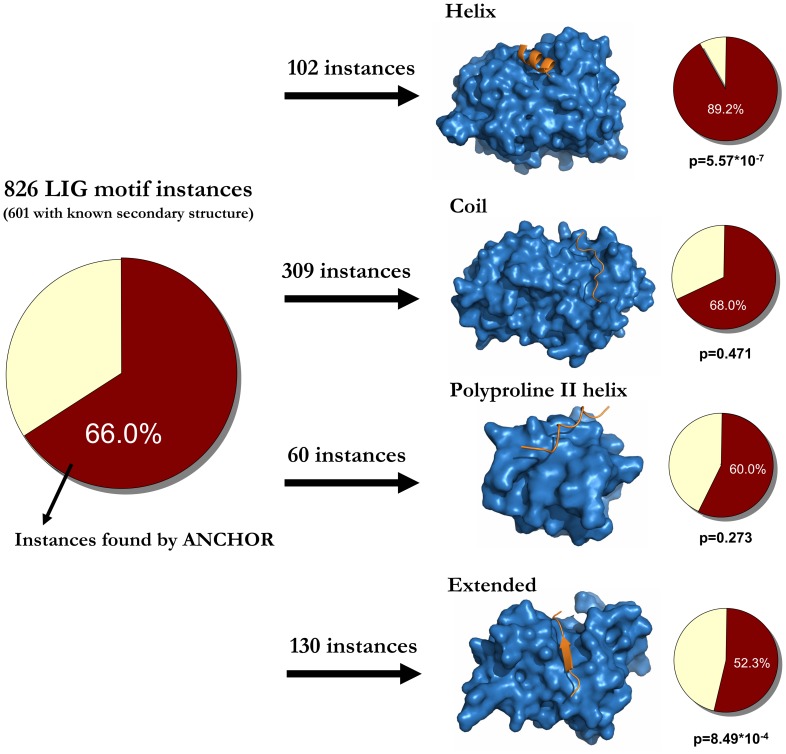
Efficiency of ANCHOR on linear motifs with respect to bound secondary structure. Motifs are classified according to the adopted secondary structure upon binding to their partner domain. The efficiency of ANCHOR for separate structural classes were calculated and were compared to the average efficiency calculated on all instances. The difference between average and secondary structure-specific efficiencies were compared using standard χ^2^ test. The resulting p-values are quoted for all 4 separate structural classes.

To gain a deeper insight into the relationship between ANCHOR predictions and linear motifs, the efficiency was evaluated for the four secondary structure classes separately. There are differences between the efficiency of ANCHOR over the four structural classes. Figure 6 shows the number of instances belonging to each of the four structural classes together with the fraction of these instances recognized by ANCHOR predictions. Helical motifs are found significantly better than average with a recovery rate of 89%. These motifs usually interact via a hydrophobic surface patch that attaches to a complementary hydrophobic groove on the partner domain. This sequence signal is readily picked up by ANCHOR and thus these instances are easily identified. Motifs binding in an irregular structure (coil) utilize a variety of binding mechanisms and the success of ANCHOR varies accordingly. As the majority of motifs belong to this class, the efficiency of ANCHOR on coil motifs is close to the overall efficiency. Proline-rich motifs that adopt a poly-proline conformation upon binding (SH3, EVH1 and WW interacting motifs) represent a special case of binding. In these cases, the efficiency was somewhat lower than average, however this difference is not significant (see figure 6). The lowest recovery rate is achieved in the case of motifs that adopt an extended structure. These proteins usually bind to their partners via beta-augmentation where the motif containing region of the protein forms an additional beta strand to an existing beta strand of the partner protein. The low efficiency of ANCHOR in this group of motifs is dominated by the extremely low recovery of motifs belonging to any of the three PDZ interacting motif classes. 39 out of the 130 instances in the extended class belong to PDZ binding motifs and the overall efficiency on these instances is only 26% (with 10 successful predictions). This low recovery is due to the fact that most of these PDZ binding motif instances reside in a structured sequential environment. They are exclusively found at the C-terminus of proteins and in many cases the interacting motif is preceded by a structured domain, such as a SAM or an amidohydrolase domain, a coiled coil region, or domains with unknown function. ANCHOR uses IUPred to identify regions that are disordered in isolation but can become ordered upon binding. This disordered prediction is hindered by the lack of flanking disordered regions and hence the returned ANCHOR score remains low. However, omitting the PDZ binding motifs from the calculations, the efficiency of the recovery of extended motifs increases to 64%.

### Examples

**Figure 7:**
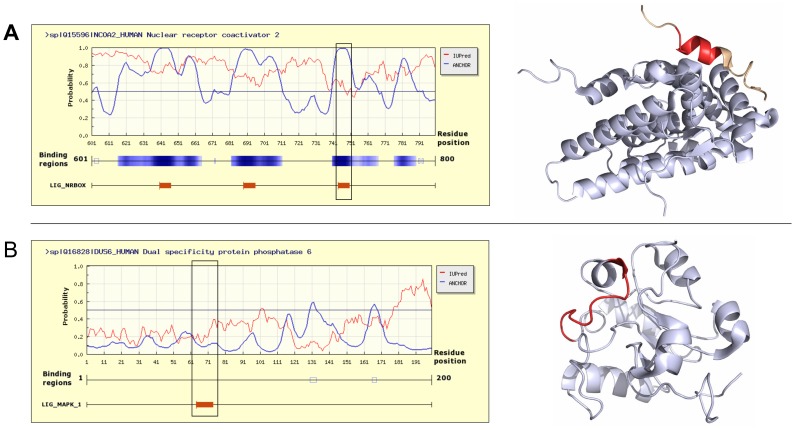
Examples of true motif instances with ANCHOR predictions. A: Three instances of the nuclear receptor binding motif (LIG_NRBOX) in the human nuclear receptor coactivator 2 protein (NCOA2). Left: IUPred (red) and ANCHOR (blue) predictions for the 601–800 region of NCOA2. Red bars mark the motif instances with the black box showing the instance for which the corresponding bound structure is shown. Right: the structure of NCOA2 (salmon) with the motif shown in red bound to the glucocorticoid receptor (grey) (structure 1 m2z). B: MAP kinase binding motif (LIG_MAPK_1) in the rhodenase domain of the human DUS6 protein. Left: IUPred (red) and ANCHOR (blue) predictions with the red bar and black box indicating the position of the motif. Right: the structure of DUS6 in monomeric form (structure 1 hzm) with the motif shown in red.

The majority of known linear motifs reside in a disordered protein region to make the interacting segment accessible for the partner molecules (see [38] and figure 5). One such example is show in figure 7A for the nuclear receptor binding motif NRBOX in the human nuclear receptor coactivator 2 protein (NCOA2). NCOA2 is a 1,464 residue long transcriptional coactivator for steroid receptors and nuclear receptors. Its dysfunction has been linked to acute myeloid leukemias. The protein contains three verified instances of the NRBOX motif through which it can bind to the human NR3C1 glucocorticoid receptor. The motifs reside in the unstructured regions of the NCOA2 protein between residues 641–882. The motif consists of three leucine residues and this hydrophobic sequence signal is readily picked up by ANCHOR and the motif regions are correctly predicted as disordered binding regions. Figure 7A also shows the known structure of one of these motif instances bound to a glucocorticoid receptor [39].

Although in fewer numbers, there are examples of biologically functional motif instances that are found inside structured domains. An example is shown in figure 7B: the MAP kinase binding motif of the human DUS6 protein. DUS6 is a 381 residue long protein implicated in various signaling pathways, including apoptosis, growth and cell speciation. It consists of two structured domains, a rhodanese and a tyrosine-protein phosphatase domain, connected by a linker region. The motif region is in a surface accessible part of the rhodenase domain and therefore can be bound by the target kinase. However, as the monomeric structure shows in figure 7B, the motif region is structured even without the presence of the binding partner [40]. As the identification of linear motif instances with ANCHOR relies heavily on the presence of protein disorder, these motifs cannot be identified with ANCHOR. This motif has an ordered structural context, where the performance of ANCHOR is very low (see figure 5). The identification of motif instances similar to these calls for the application of domain and accessibility predictions.

### Application to Whole Proteome Scans

**Figure 8:**
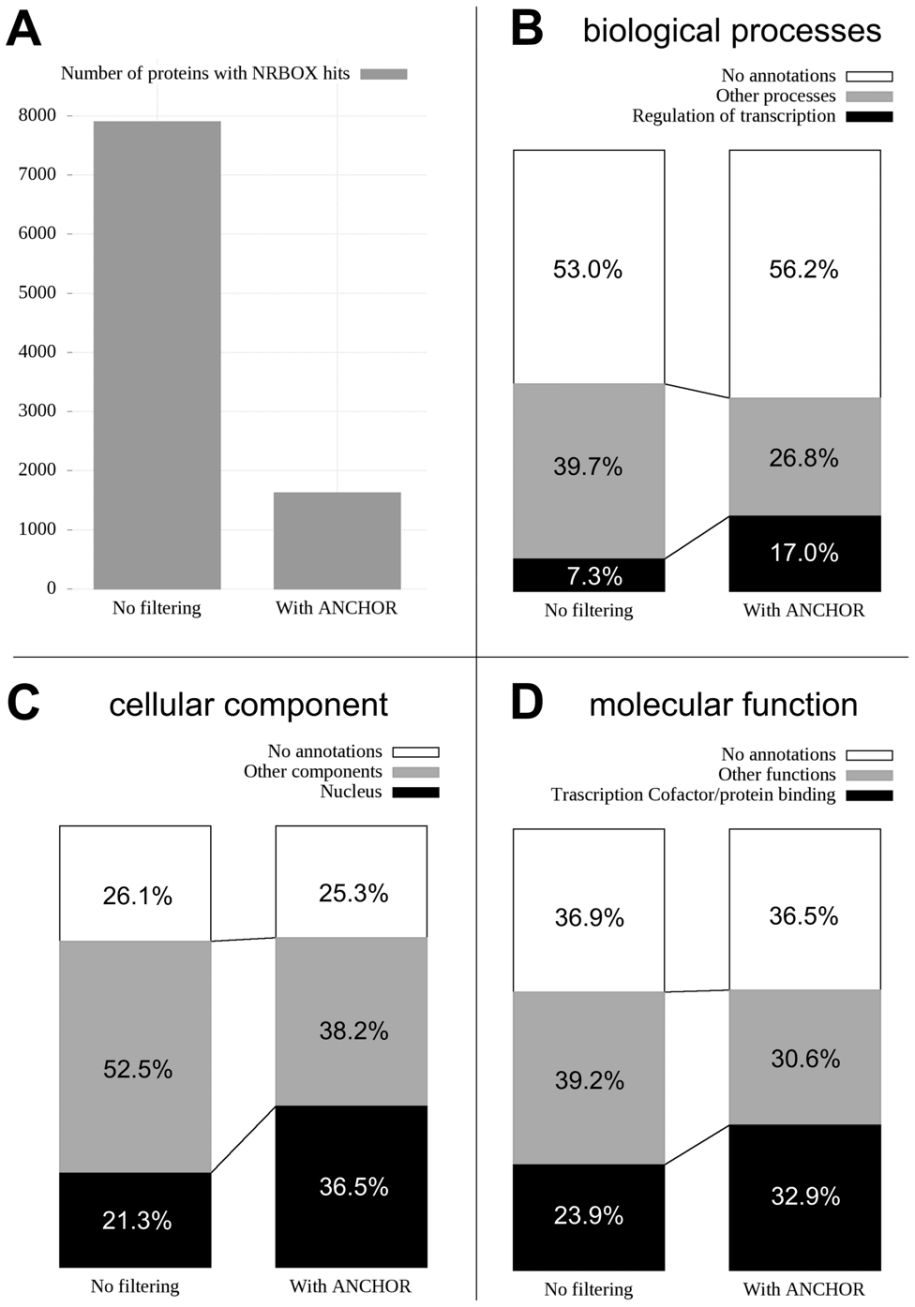
Application to whole proteome scans. Results of applying ANCHOR as a filter for scanning the human proteome for instances of the nuclear receptor interacting motif (LIG_NRBOX). A: number of proteins matching the motif; B–D: fraction of proteins containing NRBOX matches with biological process, cellular component and molecular function GO annotations (B, C and D, respectively) matching the annotations of true NRBOX instances (black boxes), with other annotations (grey boxes), and no annotations (white boxes). The height of bars in B–D represent 100% of all found motifs and thus in each sub-figure the complete left bar stands for 7,897 proteins and the complete bar on the right stands for 1,623. The two different number of hits are scaled to accurately represent enrichments of correctly annotated proteins.

To test the usability of ANCHOR in a large scale scenario, we scanned the human proteome for the nuclear receptor binding motif LIG_NRBOX and applied the ANCHOR filtering to the resulting motif hits. For NRBOX motifs the efficiency of ANCHOR is 100% on known instances with all 22 known true motifs overlapping predicted binding regions. In total 7,897 of the scanned proteins match the NRBOX motif at least once, accounting for roughly 39% of all human proteins. The number of proteins containing motif matches is reduced to 1,623 (8%) after applying ANCHOR filtering (see figure 8A).

NRBOX motifs are annotated with Gene Ontology (GO) terms from all three existing categories (biological process, cellular component and molecular function). Proteins with both unfiltered and filtered NRBOX motif matches were grouped according to their GO annotations (see Methods). In the case of all three annotation types (biological processes, cellular components and molecular functions), ANCHOR filtering increased the ratio of proteins matching the annotations of NRBOX motifs 1.4–2.3 fold (see figure 8B–D). In all three cases, the number of proteins bearing no annotations at all was high and did not change significantly due to the filtering. This shows that the relatively low ratio of proteins with correct annotations even after filtering is a consequence of the generally poor GO annotation of human proteins. Furthermore, proteins can participate in several processes, can perform multiple functions and can have multiple localizations. As a result, the proteins with annotations not matching those of NRBOX proteins are not necessarily false positives. Due to these limitations, the ratios of proteins with correct GO terms in themselves are not indicative. However, the significant enrichment of these proteins as a result of ANCHOR filtering shows that the filtering procedure greatly increases the ratio of correct motif hits while reducing the total number of hits by 80%.

## Discussion

Many vital protein-protein interactions in regulation and signaling processes are not mediated by folded domains but by relatively short stretches of amino acids that bind their target protein in a largely extended structure. The two major frameworks in which these interactions are studied are the concepts of linear motifs and disordered binding regions. In this work we analyzed the complementarity of these two concepts.

Linear motifs correspond to short consensus sequence patterns distilled from proteins with a common interacting partner domain. Such regions usually reside in disordered segments which make them accessible for the interaction. One of the most attractive features of known linear motifs is that they can be readily used to investigate candidate functional sites in eukaryotic proteins, also providing information about the key residues involved in the interactions. However, from a computational point of view, the most serious obstacle is that predicted functional instances of consensus motifs are overwhelmingly dominated by false positive matches. We chose a way of demonstrating the weakly defined nature of most motif patterns based on biological considerations (figure 1). We scanned whole proteomes from all three domains of life with motif patterns from the ELM database and found no enrichment of motif matches in eukaryotic sequences even for those motifs that are expected to be specific to these sequences. This holds true even when localization signals are analyzed separately – an alarming notion that hints at the prevalence of false positive hits in these searches. Focusing on eukaryotic proteins only, most motif patterns produce an irrationally high number of matches with about 70% of motifs matching 1 out of 100 sequences and about 30% of motifs matching 1 out of 10. This high number of predicted interacting partners for domains such as WW, 14-3-3 and cyclin contradicts the known topology of protein interaction networks. Furthermore, the number of pattern matches in the three domains of life does not correlate with the presence of the target domains for individual motifs either (figure 1C). Due to the weak predictive power of linear motifs, the proteome-wide identification of functional sites remains a challenging task and the results need to be interpreted exercising proper caution [41]. Even in the case of highly specialized methods optimized for a single type of motif [42], the accuracies of motif instance prediction algorithms remain low.

To improve the predictive power, various context-based rules and filters are being developed and applied to reduce the amount of false positive matches. Most motifs are only functional in certain cellular or taxonomical context and this information can be built into the search process. Furthermore, there can be large differences in how well the regular expressions are defined. Various scoring schemes were constructed to quantify the probability of the occurrence of a given motif in random sequences to give an estimate on the expected false positive rate [29], [43], [44]. Such measures are also incorporated into the ELM server to provide warnings for the user of what order of magnitude of false positives can be expected when using only the pattern to search for true motif instances. However, these estimations do not take into account the precise correlations between the occurrences of various amino acids that is introduced by the structural context, which has a major impact on both the expected and the real number of occurrences of the motifs. The distribution of most amino acids is far from random and in certain cases this correlates heavily with structure formation. For example hydrophobic residues tend to cluster in the core regions of globular proteins (positive correlation) and prolines are likely to be part of proline rich segments (negative correlation). As the true occurrences of motifs are also coupled with structure with most linear motifs residing in disordered regions, recognizing the proper structural context is crucial.

In this work we suggest that ANCHOR – a method developed to recognize regions of disordered proteins capable of binding to an ordered partner – can be used as a structural filter to improve the predictive power of linear motifs. The basis of this is the strong correlation between disordered binding regions and linear motifs. Disordered binding regions predicted by ANCHOR overlap with known linear motifs with a significantly higher ratio than expected by random (see figure 3). Furthermore, ANCHOR is much more sensitive to true motif instances than for protein segments simply matching a motif pattern. Therefore, the combined method can be effectively used to enrich the number of true positive motif hits when scanning through unknown sequences by discarding the motif hits that do not overlap with ANCHOR predictions (figures 4 and 8). This filtering provides more reliable results as correct motifs are enriched while the total number of hits are reduced by nearly an order of magnitude (figure 4). The generality of ANCHOR, being applicable to any protein sequence is a clear advantage compared to other, commonly used filters that require the prior knowledge of the localization of the protein or the ability to identify existing domains based on sequence alignments. Furthermore, the performance of ANCHOR in motif instance identification seems to be largely independent of the adopted secondary structure in the bound form (figure 6). These results indicate that ANCHOR can be used as an effective filtering tool in motif searches.

The strong connection between linear motif-mediated binding and interactions of disordered proteins is quite remarkable, given some fundamental differences between the two models. To understand this, a closer look at the these concepts is required. It is usually assumed that linear motifs act independently of protein tertiary structure and only a few residues participate in the binding that are common between the partners. This, however, is an idealized scenario that has several limitations. Most true instances reside in partially or fully disordered protein regions, which makes them readily accessible for interactions [38], however, some true motif instances can be found in structured protein regions (see figure 5 and figure 7 for an example). In recent studies it has been shown that the evolution of motif flanking regions is not independent of the evolution of the motif itself and the residues surrounding real motif instances appear more conserved [45]. The main factors behind this was found to be the preservation of the proper structural context in which the motif is accessible and the increase of specificity by forming additional contacts, on average accounting for around one fifth of the total binding energy [46]. Our results also indicated that the local sequence elements contain additional information necessary for the binding and this information is not captured by the motifs. Furthermore, the same binding surface can be involved in multiple binding modes that can only be captured by multiple motifs. In other cases, a more refined definition of motifs can help to understand the molecular basis of different specificities within certain subclasses.

The concept of disordered binding regions approaches the binding from a different angle by focusing on the specific region of a disordered protein that becomes ordered upon the binding. Analyses of structured complexes of disordered proteins showed that the length of these regions can vary between 10 to at least 70 residues, and it is characteristically longer than a single motif. At a closer look, however, there are indications that the binding is not even, and certain residues make more significant contributions to the binding. This can be reflected by the distribution of atomic contacts, evolutionary conservation or alanine scanning. One well-characterized example is the binding of a 69 residues long segment of the disordered human p27 to the complex of cyclin A-cyclin-dependent kinase 2. Within this longer region there are individual sites with different structural and functional features. Relatively stronger atomic contacts are formed between the N-terminal regions and the two short C-terminal regions forming beta hairpins and a 3–10 helix, respectively. The intervening helical segment, that is prefomed even in the unbound form, makes fewer contacts in the complex. Both the N-terminal and C-terminal regions have important functional roles. The N-terminal region recognizes cyclin A through a motif that is shared among cyclin binding proteins. The C-terminal region is responsible for the blocking of the catalytic site of CDK2. This and other examples indicate that the binding of disordered proteins is more general and cannot be reduced to linear motifs. Nevertheless, identifying residues that have a prime role in terms of function, specificity and partner recognition within the longer segments of disordered binding regions is an important challenge in this field [47].

The differences between the concepts of linear motifs and disordered binding regions are reflected in their dedicated prediction methods. Disordered binding regions can be predicted from the amino acid sequence using ANCHOR. The method focuses on the disordered nature of these segments in isolation and their ability to undergo a disorder-to-order transition. The predictions are based on estimated interaction potentials that are averaged over several residues. As a consequence, predicted binding regions are relatively insensitive to single amino acid changes, although in reality the mutation of a single key residue can impair the binding. The incorporation of motif matching can introduce a heavy emphasis on these residues. Furthermore, the presence of motifs automatically introduces information about the interaction partner. In return, ANCHOR is able to remedy the high false positive rate of motif searches. As there is a continuous score behind ANCHOR predictions, this false positive rate is tunable to fit custom applications. On the other hand, ANCHOR incorporates the estimation of potential interaction energies, through which the disordered nature of the protein is taken into account. This way ANCHOR inherently introduces context-dependency into the predictions. Both prediction tactics have their own strengths and weaknesses and the combined approach is able to get the best of both worlds.

Altogether, our results support the complementarity of the linear motif and disordered binding region concepts. The overlap of the two approaches has strong practical implications in a wide range of fields. On one hand ANCHOR can be effectively used in filtering putative binding motifs which can aid the prioritization of candidate motifs for experimental works and improve the quality of proteome-wide systems biology analyses. This can aid the more reliable reconstruction of protein-protein interaction networks. Apart from the reliable filtering of motif hits, the combined method can also advance the distillation of new motifs from protein sequences, termed *de novo* motif discovery. The starting point in these studies is often the assembly of sequences of proteins known to have a common interacting partner. In certain cases, high resolution structures of complexes interacting via linear motifs can be exploited in the distillation of new consensus motifs [37]. In more general cases, the algorithmically most challenging step is usually the identification of the regions through which these proteins interact using the sequence alone. As consensus motifs are usually composed of only a handful of residues, this sequence signal is difficult to pick up, as generally the rest of the sequence is unrelated and presents itself as noise. Different approaches can aid the more reliable identification of new motifs [44], [48], [49] (for a recent review about motif discovery see [50]), including masking of poorly conserved residues [51] and taking biological context into account [45], [52]. The strong correlation between ANCHOR predictions and biologically relevant motif instances gives way to restricting the protein regions to consider when searching for the interacting regions. As ANCHOR takes into account all residues in the sequence when marking potential binding regions, the presence of less conserved flanking residues can be turned to signal from noise.

In general, the combination of the two predictions corresponding to the two binding models enables us to get the advantages of both approaches: predict interactions with relatively low false positive rate, with structural context and with information about the partner. Furthermore, the integration of the two concepts is also necessary for a deeper and a more complete picture of the molecular details of protein-protein interactions.

## Methods

### Databases

#### Motif patterns and instances

Linear motif patterns and instances were taken from the ELM website (newest release as of Oct. 12, 2011). In total 166 motif patterns were found categorized into four distinct groups (CLV - cleavage sites: 8 motifs; LIG – ligand binding sites: 107 motifs; MOD - modification sites: 30 motifs; TRG – target sites: 21 motifs). Instances of the LIG motifs were checked and filtered and only ones with “true positive” logical instance annotations were kept. This checking reduced the number of instances from 1,117 to 1,051. Furthermore, motif instances residing in highly similar sequences were removed with only one representative being kept. Similarity filtering was done with BLAST using a 10^−4^ e-value cutoff. All protein sequences containing annotated instances were input to an all-against-all BLAST search. Based on local similarity of the motif containing region of proteins, clusters of similar instances were created and one representative was chosen at random while the rest of the instances were omitted in further analyses. Multiple instances from the same sequence were only kept if they did not produce a significant similarity using the above criteria. ELMs that do not have any annotated instances were also omitted. As a result 826 instances were kept encompassing 97 ligand binding motifs.

#### Eukariotic, bacterial and archaeal SwissProt datasets

SwissProt sequences were retrieved through the Uniprot ftp server (ftp.uniprot.org) on Nov. 04, 2011. Eukaryotic sequences that constitute the Eukaryotic SwissProt database were assembled by joining the appropriate available taxonomic divisions (fungi, plants, vertebrates, invertebrates, mammals, rodents and human). The resulting database contains 171,208 sequences. Bacterial and archaeal datasets were retrieved from the corresponding taxonomic divisions of the Uniprot server. These datasets contain 326,910 and 18,674 sequences.

#### Human proteome dataset

Human protein sequences were downloaded from the appropriate taxonomic division from the Uniprot ftp server on Nov. 04, 2011. The database contains 20,256 sequences.

#### Identifing bound structures of motif instances

Bound structures of motif instances were collected from the ELM database and additional structures were identified using a BLAST search. The sequence of each instance protein of each LIG motif was used as a query sequence in a BLAST search against the PDB. Significant hits were collected and filtered. Only those matches were kept that had one chain matching the motif containing segment of the instance (at least 90% sequence identity, excluding his-tags and other engineered parts) and where this region was in contact with another protein partner.

#### Secondary structure assignment of bound instances

For each bound structure the secondary structure of the motif containing protein was calculated using the DSSP and PROSS algorithms on a per residue basis. The overall secondary structure type of the bound segment was determined by the majority of the conformation of the amino acids belonging to the motif region and being in contact with the partner.

#### Pfam domains

The number of PCNA, PDZ and cyclin domain occurrences in the sequences from the three domains of life were collected from the Pfam database (http://pfam.sanger.ac.uk/).

### Prediction Methods

#### IUPred

The default version of IUPred was used (http://iupred.enzim.hu) with the “long” setting.

#### ANCHOR

We used the default version of ANCHOR (http://anchor.enzim.hu), but lowered the cutoff value to 0.4 for disordered binding regions. However, we kept both included filters, meaning that all predicted binding regions shorter than 6 residues and predicted binding regions with extremely low disorder scores were removed. We considered an ELM instance found if there was an overlap between the instance and a binding region predicted by ANCHOR.

### Statistical Analyses

#### Assessing the significance of overlap between motif instances and ANCHOR regions

The expected overlap between ANCHOR regions and randomly selected protein segments was determined in a stepwise fashion. First, 10,000 regions of length *l* were selected randomly from the sequences of the UniRef50 non-redundant database. These sequences were input to ANCHOR and the fraction of randomly selected segments overlapping with ANCHOR predicted regions were calculated. This procedure was repeated 10 times and the average overlap % was calculated. This was done with varying the *l* length between 3 and 20. From this the probability *p* of a randomly selected segment of length *l* overlapping with ANCHOR regions was fitted: 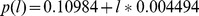. The significance of the overlap between real motif regions and ANCHOR was calculated using the binomial distribution using *p*(*l*) as the background probability, substituting the average length of the known instances of each motif. The overlap was considered significant, if the probability of the overlap based on the random case was below 0.01.

### GeneOntology (GO) Annotations

GO annotations of the inspected LIG_NRBOX motif was taken from the ELM website. These annotations include from all three main categories of GO (biological process, cellular component and molecular function). From the biological processes the “Regulation of transcription” (GO:0006355) was kept, as the other annotated term (“Positive regulation of transcription”, GO:0045893) is a direct child term of GO:0006355. From the molecular function annotations the “Transcription Co-activation” (GO:0003713) was also omitted due to being a child term of “Transcription Cofactor” (GO:0003712). The “Transcription Factor Binding” (GO:0008134) term was replaced with its ancestor term “Protein binding” (GO:0005515).

GO annotations of human proteins were taken from the Gene Ontology Annotation section of the EBI homepage (http://www.ebi.ac.uk/GOA/proteomes.html). These annotations were mapped to the higher level annotations given in the Generic GOslim subset of GO. However, to remove bias in the analysis, Generic GO terms were slightly modified. All root level terms were removed (biological_process, cellular_component and molecular_function) in order to remove the excessive but uninformative term hits. For similar reasons very broad cellular component terms (“cell”, “intracellular” and “organelle”) were also excluded. The biological process term “Regulation of biological process” (GO:0050789) was removed as it is not used in the EBI human proteome annotations. Instead, its child term “Regulation of transcription” was added. Furthermore, the molecular function term “Transcription Cofactor” was also added as none of its child or ancestor terms are included in the Generic GOslim.

## Supporting Information

Table S1 **Secondary structure classification of true LIG motif instances.** Secondary structure classification was based on bound structures of true positive instances. Structure assignation was done with the DSSP and PROSS algorithms: H – helical, E – extended (β structures), C – irregular, P – poly-proline II helix. Column 4 shows the ID of respective PDB entry together with the chain ID of the protein containing the motif, then the interacting domain partner, separated by ‘-’.
(XLS)

Table S2 **Secondary structure classification of LIG motif classes.** Secondary structure classification was based on representative bound structures of true positive instances. Structure assignation was done with the DSSP and PROSS algorithms: H – helical, E – extended (β structures), C – irregular, P – poly-proline II helix.
(XLS)
